# Climate‐Induced Saltwater Intrusion in 2100: Recharge‐Driven Severity, Sea Level‐Driven Prevalence

**DOI:** 10.1029/2024GL110359

**Published:** 2024-11-22

**Authors:** Kyra H. Adams, J. T. Reager, Brett A. Buzzanga, Cédric H. David, Audrey H. Sawyer, Benjamin D. Hamlington

**Affiliations:** ^1^ Jet Propulsion Laboratory at California Institute of Technology Pasadena CA USA; ^2^ School of Earth Sciences Ohio State University Columbus OH USA

## Abstract

Saltwater intrusion is a critical concern for coastal communities due to its impacts on fresh ecosystems and civil infrastructure. Declining recharge and rising sea level are the two dominant drivers of saltwater intrusion along the land‐ocean continuum, but there are currently no global estimates of future saltwater intrusion that synthesize these two spatially variable processes. Here, for the first time, we provide a novel assessment of global saltwater intrusion risk by integrating future recharge and sea level rise while considering the unique geology and topography of coastal regions. We show that nearly 77% of global coastal areas below 60° north will undergo saltwater intrusion by 2100, with different dominant drivers. Climate‐driven changes in subsurface water replenishment (recharge) is responsible for the high‐magnitude cases of saltwater intrusion, whereas sea level rise and coastline migration are responsible for the global pervasiveness of saltwater intrusion and have a greater effect on low‐lying areas.

## Key Points

- First global analysis of future saltwater intrusion vulnerability responding to spatially variable recharge and sea level rise is provided
- Recharge drives the extreme cases of saltwater intrusion, while sea level rise is responsible for its global pervasiveness
- Nearly 77% of global coastal areas below 60° north will undergo saltwater intrusion by 2100

## 1 Introduction

**Figure 1:**
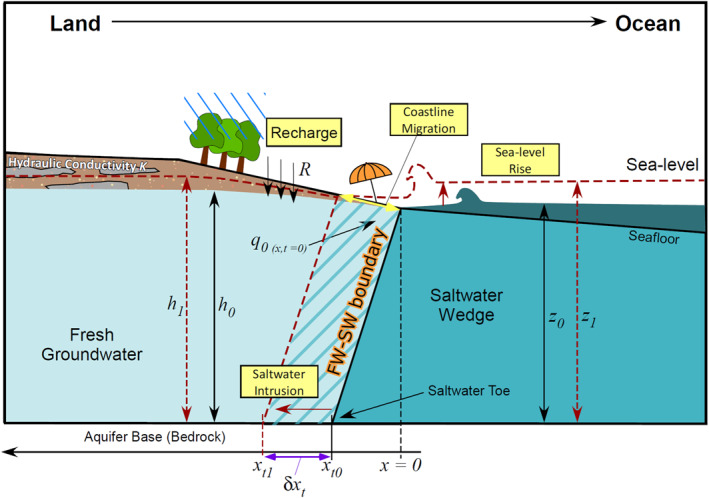
Cross‐sectional schematic diagram of a coastal aquifer undergoing saltwater intrusion. Aquifer base (bedrock) is the bottom of the figure. The aquifer is recharged onshore at a rate *R*. Fresh groundwater discharges directly to the ocean with a flux of *q*_0,_ pushing against the saltwater wedge and defending against salinization. FW‐SW (freshwater‐saline water) boundary denotes the fresh‐saline groundwater interface. The most landward point of the FW‐SW boundary along the aquifer base is the saltwater toe (*x*_*t*0_; under script _*t*0_ denotes “toe” at time = 0). This toe moves from *x*_*t*0_ to *x*_*t*1_ (δ*x*_*t*_) with sea‐level rise (red dotted line, *z*_0_ to *z*_1_), salinizing fresh groundwater (dashed areas) and migrating the coastline (yellow line). If given topographic space (i.e., a thick unsaturated zone), hydraulic head will also rise (*h*_0_ to *h*_1_; see more under Text S2 in Supporting Information S1: Boundary conditions and Figure S3 in Supporting Information S1).

Saltwater intrusion is the movement of saline seawater into coastal freshwater areas due to rising seas and changing coastal hydrology. Saltwater intrusion has increasingly emerged as a concern in coastal regions due to its impacts on freshwater ecosystems and underground urban infrastructure (Abdelhafez et al., 2022; Ferguson & Gleeson, 2012; Michael et al., 2017; Setiawan et al., 2022; Tansel & Zhang, 2022; White & Kaplan, 2021). Driven by climatic changes in sea level and the water cycle (Michael et al., 2013; Werner & Simmons, 2009), saltwater intrusion (narrowly defined within this work as the subsurface lateral intrusion of seawater; Figure 1) is difficult to quickly assess at large scales compared to the salination of surface water bodies due to its below‐ground occurrence and monitoring challenges. Yet, the extent of future saltwater intrusion will be precariously controlled by both terrestrial and oceanic forces that depend on climate, including aquifer recharge on‐land and sea level rise offshore. Thus, a vulnerability assessment that encompasses both changes, with considerations to the unique coastal setting at hand, is needed.

While many coastal regions around the world are starting to witness the impacts of saltwater intrusion, future saltwater intrusion driven by climatic changes has not yet been assessed globally. Regional studies are able to leverage numerical modeling tools, geophysical techniques, and traditional groundwater sampling techniques (e.g., Abd‐Elaty & Zelenakova, 2022; Befus et al., 2020a; Jasechko et al., 2020a; Meyer et al., 2019; Roy & Datta, 2020). However, no global assessments have been put forth that integrate the critical climatic pressures already in motion across the land‐ocean boundary. Recent advancements in future sea level projections clearly indicate strong regional variations, with some regions even experiencing sea level retreat (Adhikari et al., 2019; Hamlington et al., 2020; IPCC, 2021; Nerem et al., 2018, 2022; Widlansky et al., 2020). However, the few existing regional‐to global‐scale analyses for saltwater intrusion have neglected spatially variable sea level rise (Befus et al., 2020; Ferguson & Gleeson, 2012; Michael et al., 2013). A recent study incorporated climate scenario variability in sea level rise projections, but did not explicitly account for future recharge changes that can outweigh the effects of sea level rise on saltwater intrusion (Zamrsky et al., 2024). Furthermore, the study quantified changes in the volume of fresh groundwater reserves, which are important for coastal water security but less relevant to understanding damage to croplands (Gopalakrishnan et al., 2019) or to subsurface infrastructure, including building foundations, tunnels, and stormwater and septic systems (Habel et al., 2024). In these applications, a more relevant metric is the landward movement of the freshwater‐saltwater interface.

To this end, we provide a new global assessment of saltwater intrusion using an ensemble of state‐of‐the‐art recharge and sea level projections, considering the unique geologic and topographic setting of coastal watersheds in our calculations. Saltwater intrusion as a process can have nuances in its progression, contingent on the interactions between the groundwater table and topography defining its boundary conditions (Michael et al., 2013; Werner & Simmons, 2009), and coastline migration from inundation as sea level rises (Ataie‐Ashtiani et al., 2013; Ketabchi et al., 2016). While we cannot account for complex local geologic heterogeneity at this scale (e.g., Zamrsky et al., 2024), we make the important step of considering the two aforementioned processes within our calculations, as well as incorporating the best globally available data set for hydraulic conductivity. The presented model also does not include pumping and other local to regional processes, but rather focuses on the large climatic drivers of saltwater intrusion. The value of this work lies within its ability to integrate the best available global data sets, spanning from aquifer properties, climatic projections, to modeled ocean density based on remote sensing data without the need for intensive modeling, yielding a spatial pattern of vulnerability that can further guide more regionalized investigations utilizing more detailed tools. Further, its agility in incorporating different recharge or sea level rise projections according to user interest allows for quick comparisons across future scenarios.

## 2 Data and Methods

This study leverages 1‐D analytical equations (Bear et al., 1999; Custodio, 1987; Strack, 1976; Werner & Simmons, 2009) to estimate future saltwater intrusion vulnerability across 60,638 coastal watershed locations spanning the global coastline. Geographic information for the catchments (e.g., distance from the present day coastline to the hydraulic divide) was compiled through analysis of information presented in HydroSHEDS (https://www.hydrosheds.org/; Hydrological data and maps based on Shuttle Elevation Derivatives at multiple Scales; Lehner et al., 2008). Analysis focused on locations below 60° north latitude where water basin delineation data are available and most of the world's population resides.

Coastal watersheds were delineated and their seaward groundwater discharge rates were estimated (Figure 1; *q*_0_) following the field‐verified methods of Sawyer et al. (2016) and Zhou et al. (2019), which assumed that the only groundwater input to each catchment is the net recharge rate adjusted for evaporative losses, *R* [l/*t*], and that the only groundwater output is the oceanward fresh groundwater flux [l^2^/*t*] per unit length of shoreline (see Figures S1 and S2 in Supporting Information S1; readers are directed to the aforementioned references for more information). Coastal catchments were selected such that none contained any mapped streams present within the HydroSHEDS data set, which globally represents rivers that have upstream contributing areas greater than 8 km^2^ (Figure S1 in Supporting Information S1). Through this constraint, the analyzed watersheds were ensured to export groundwater to the coast rather than a mapped stream, as the estimation of saltwater intrusion near streams must rely on more complex numerical methods to account for drainage toward multiple boundaries (Werner, 2017; Werner & Simmons, 2009). Further, we considered that coastal catchments with fresh groundwater discharge fluxes below the analytically resolvable threshold were considered to be fully intruded up to the hydraulic divide or landward boundary of the catchment (Werner, 2017).

Aquifers in the selected watersheds were assumed to be unconfined, with isotropic and homogeneous hydraulic conductivities. We did not limit the analysis to specific rock types (e.g., unconsolidated sediment vs. fractured bedrock). While it is expected that saltwater intrusion would progress at various rates in layered aquifer systems, results here focus on the uppermost unconfined aquifer in coastal regions that would be most societally relevant and has been shown to be the most influenced by climatic changes (Meyer et al., 2019). For such aquifers under steady‐state conditions, the horizontal distance from the shoreline to the most landward point (toe) of the FW‐SW interface (Figure 1; *x*_*t*_) can be estimated from the Ghyben‐Herzberg and Dupuit‐Forchheimer approximations (Custodio, 1987; Werner & Simmons, 2009), with modifications to account for coastline migration and associated inundation (*M*; see Text S1 in Supporting Information S1):

(1) $$x_{t}=\frac{q_{0}}{R}-\sqrt{{\left(\frac{q_{0}}{R}\right)}^{2}-\frac{K\left(1+\alpha \right)z_{0}^{2}}{R\left(\alpha^{2}\right)}}+M$$

where *q*_0_ is freshwater flux, *R* is recharge rate, *K* is hydraulic conductivity, *α* is a density ratio for fresh and saline groundwater ($a=\frac{\rho_{f}}{\left(\rho_{s}-\rho_{f}\right)}$), *z*_0_ is present‐day depth from the base of the aquifer to sea level, and coastline migration is *M*. Lateral saltwater intrusion was calculated as the difference between current and future *x*_*t*_ values when 2100 recharge, sea level, or coastline migration were introduced. For each location, the calculations also considered the relationship between expected groundwater table rise and mean watershed elevation (also isolated from HydroSHEDS) to determine the appropriate boundary condition and analytical calculation method (see Text S2 in Supporting Information S1).

For each spatially variable parameter (*K*, *R*, *a*, *z*), a representative value for the watershed was isolated by selecting the value at the centroid of the given watershed from the spatial data set employed. Aquifer thickness was gleaned from Zamrsky et al. (2018) which provides estimates for unconsolidated regions, with authors noting its sufficiency for large‐scale aquifer modeling. Hydraulic conductivity was isolated from GLHYMPS (Global Hydrogeology Maps; Gleeson et al., 2014). When values were unavailable due to lack of data at that exact location, the mean value of that parameter for data‐available regions was checked against previously utilized ranges in literature and used if reasonable (e.g., for aquifer thickness, hydraulic conductivity), or the nearest spatial data pixel was selected instead (e.g., for recharge). Seawater density was obtained from the ocean model ECCO v4r4 (Estimating the Circulation and Climate of the Ocean; Carroll et al., 2020) 2017 annual mean outputs, and to the best of our knowledge, is the first attempt at spatially varying the coastal ocean density in such calculations although it plays a relatively minor role in saltwater intrusion variability compared to other parameters (Text S3 in Supporting Information S1). Recharge (groundwater infiltration) for current conditions was calculated using outputs from NASA's Global Land Data Assimilation System (GLDAS) Version 2.1 (Rodell et al., 2004) by analyzing mean VIC (Variable Infiltration Capacity) and NOAH (**N**ational Centers for Environmental Prediction‐**O**regon State University‐**A**ir Force‐**H**ydrologic Research Lab) model outputs spanning two decades (2000–2020). Future recharge was obtained from ISIMIP (Inter‐Sectoral Impact Model Intercomparison Project) protocol 2b (Frieler et al., 2017) outputs using the CLM4.5 (Community Land Model 4.5) model with GFDL‐ESM2M (Geophysical Fluid Dynamics Laboratory‐Earth System Model 2M) climate forcing (Dunne et al., 2012, 2013) and EWEMBI bias adjustments (E2OBS, WFDEI and ERAI data Merged and Bias‐corrected for ISIMIP; Lange, 2016). The mean recharge for climate scenarios rcp26 and rcp85 for the years 2090–2100 were calculated as estimates of future recharge. For sea level rise, regional projections developed as part of the Intergovernmental Panel on Climate Change Sixth Assessment Report were used. These projections include the six major driving components of sea level change: changes in the Antarctic ice sheet, Greenland ice sheet, glaciers, land water storage, ocean dynamics (including thermal expansion) and vertical land motion. We consider SSP scenarios 2–4.5 and 5–8.5, which correspond to middle of the road and intensive fossil‐fuel development, respectively (IPCC, 2021). The sea level changes result from processes whose projections are at least medium confidence, except for two low‐confidence process contributions in 5–8.5 that account for the low‐likelihood but high impact ice‐sheet changes that cannot be ruled out.

## 3 Quantifying Future Vulnerabilities to Climate‐Driven Saltwater Intrusion

We project saltwater intrusion estimates for the year 2100 utilizing the methods described above. Both recharge and sea level rise are projected to be highly spatially variable in 2100, with some regions even predicted to have little or negative sea level change due to glacial isostatic adjustment (e.g., Hudson Bay) (Adhikari et al., 2019; Figure S4 in Supporting Information S1). While it is not a linear additive process between recharge and sea level rise driving saltwater intrusion, three separate calculations are provided to represent the individual effects of recharge and sea level rise on saltwater intrusion: (a) Case A: Changing only recharge to 2100; (b) Case B: Changing only sea level to 2100; and (c) Case C: Changing both to 2100.

**Figure 2:**
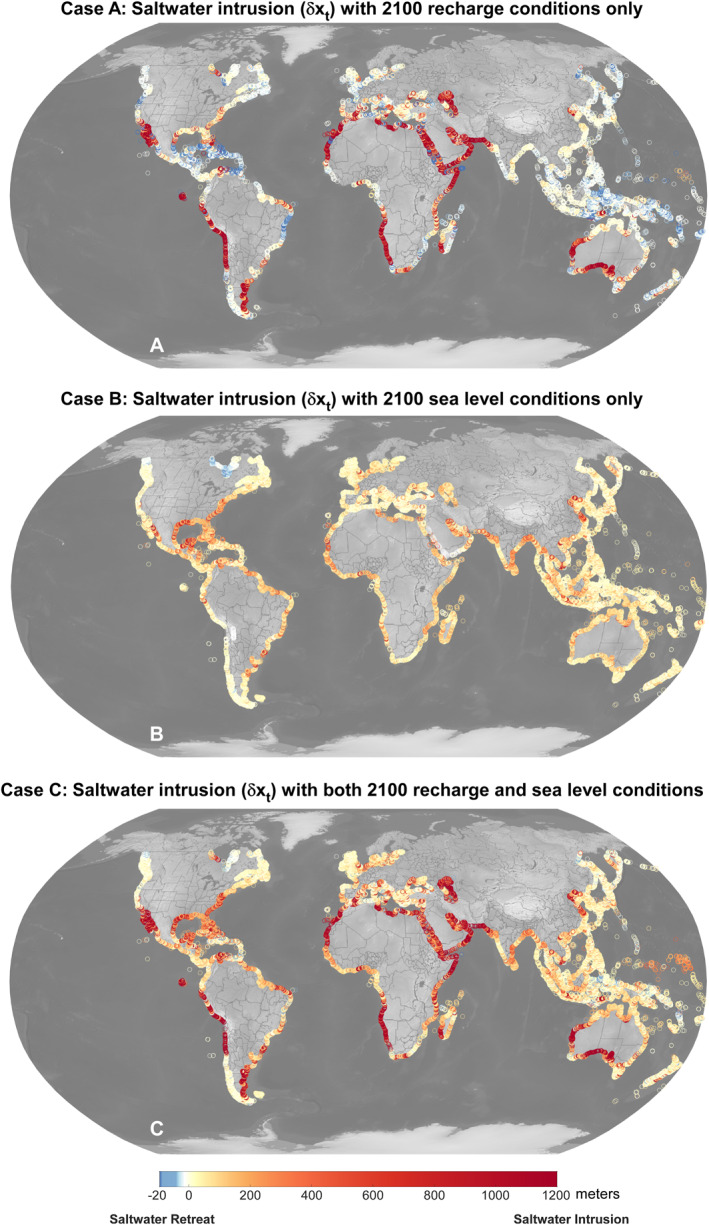
(a) Saltwater intrusion (δx_t_) relative to present‐day coastline with 2100 recharge (Case A), (b) 2100 sea‐level (Case B), and (c) both (Case C). Changes to recharge drive high magnitudes of saltwater intrusion, while sea level drives it to be much more pervasive globally. With sea level rise, previously less vulnerable regions of high recharge (e.g., Southeast Asia) emerge as more vulnerable regions due to its low‐lying topography.

In Case A, the spatial patterns of saltwater intrusion closely track climate‐driven recharge changes, with relatively dry regions of already low recharge (e.g., Arabian Peninsula, West Australia) experiencing the gravest saltwater intrusion (Figure 2a). 45% of the watersheds experience positive and significant intrusion (>1 m), while 42% undergo “saltwater retreat” from increased recharge. While regions that experience analytical saltwater retreat are not expected to freshen instantaneously, they represent regions that would have a larger freshwater body at equilibrium and would be less vulnerable to recharge‐driven impacts. The remaining watersheds show no significant change to the current saltwater toe position (<1 m). While here we only consider climate‐driven recharge changes, these results can be used to infer general saltwater intrusion behavior should a region experience additional changes to the coastal freshwater budget through other pathways (e.g., groundwater extraction).

In Case B (Figure 2b; changes to sea level), though the globally averaged saltwater intrusion is less than Case A (281 m in Case A vs. 64 m in Case B; retreat excluded in the global average), saltwater intrusion is much more spatially pervasive, with 82% of watersheds experiencing intrusion. While sea level rise is not responsible for the most extreme scenarios of saltwater intrusion (Case A), it drives more extensive changes across the global coast. Case B also suffers the compounding effects of coastline migration from overland flooding (which logically are not included in Case A where only recharge changes; see Text S1 in Supporting Information S1). Low‐elevation regions, such as Southeast Asia, are particularly vulnerable to these sea level rise effects. In some of these areas, the climate is expected to become wetter (Case A), resulting in opposing forces. Regions such as Alaska, British Columbia, and Hudson Bay notably had saltwater retreat responding to falling relative sea level. This highlights the ability of the approach to capture previously missed nuances in sea level change variability across the globe.

With effects of both recharge and sea level rise (Case C; Figure 2c), nearly 77% of watersheds experience saltwater intrusion in 2100, with a mean lateral movement of about 210 m. Results emphasize that fresh groundwater aquifers in a majority of coastal watersheds will suffer climate‐driven impacts of salinization, with recharge driving much of the extreme cases of saltwater intrusion, and sea level rise and conjunctive coastline migration being responsible for the pervasiveness of saltwater intrusion.

These results across model Cases A‐C are specific to regions in the mid‐ and low‐latitudes and exclude northern high latitudes where permafrost may occur. Sea level will fall in many high‐latitude regions beyond our analysis, due to gravitational effects and glacial isostatic adjustment, thus we expect that including these regions would decrease the global statistical mean of saltwater intrusion. Further, the results provide baseline expectations for the large‐scale spatial patterns of saltwater intrusion, and other processes more relevant to local or regional settings (e.g., pumping, geologic heterogeneity, vertical land motion, episodic events) should be considered in yielding more contextualized predictions within specific locations.

## 4 Importance of Hydrogeologic Characteristics in Saltwater Intrusion Estimations

**Figure 3:**
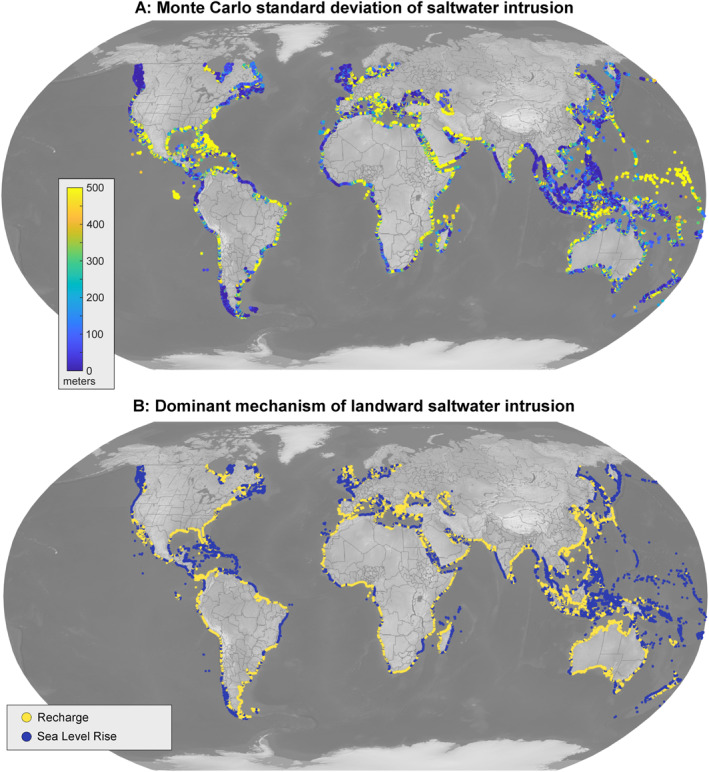
Sensitivity of saltwater intrusion projections to parameter uncertainty and dominant drivers by region. (a) Standard deviation of saltwater intrusion from 100,000 iterations of Monte Carlo simulations quantifying sensitivity to hydraulic conductivity, aquifer thickness, seawater density, groundwater recharge, and sea level rise. The standard deviations were weakly correlated with hydraulic conductivity and aquifer thickness. (b) Dominant mechanism of landward saltwater intrusion. Only landward migrations of the saltwater toe (intrusion, and not seaward retreat) were considered in comparing saltwater intrusion induced by recharge change or sea level rise (which includes coastline migration).

Saltwater intrusion sensitivity to hydraulic conductivity, aquifer thickness, seawater density, groundwater recharge, and sea level rise was quantified using 100,000 iterations of Monte Carlo simulations for each watershed. First, we estimated current saltwater toe locations using current recharge and sea level rise conditions and the watershed's unique aquifer parameters. Then, we used randomly selected values from uniform distributions for seawater density, recharge, and sea level rise and log normal distributions for hydraulic conductivity and thickness over defined parameter ranges to calculate future saltwater toe location 100,000 times. Lateral intrusion was calculated as the difference between initial and final toe location, and the standard deviation of the resulting intrusion was calculated for each watershed (Figure 3a; see Text S3 in Supporting Information S1). Uncertainty of saltwater intrusion estimates tends to be greatest in regions such as the Florida peninsula, parts of the Arabian Peninsula, and the Pacific islands. These regions have high hydraulic conductivity, thick aquifers, or low recharge under “current conditions”, indicating that such factors may drive higher uncertainty for saltwater intrusion. The Pearson correlation coefficient between saltwater intrusion uncertainty and conductivity was the highest, at *r* = 0.44, closely followed by thickness at *r* = 0.33. Thus, geologic uncertainty poses the greater limits to accurate estimation of saltwater intrusion within the chosen model approach and motivates the need for continued refinement to standardized global hydrogeologic data sets. Werner and Simmons (2009) similarly noted the potential for uncertainties due to geologic heterogeneity, as well as temporal variability in the forcing factors like sea level rise and recharge, which together influence the width of the transition zone from fresh to saline groundwater. Thus, both model choice (dimensionality and assumptions) and parameter choice (hydraulic conductivity, aquifer thickness) contribute to uncertainty about saltwater intrusion (Dell’Oca et al., 2020). A comparison of model frameworks for estimating saltwater intrusion is an important area for future work. While geologic representations were simplified for the purposes of this work, it is expected that at local or regional scales, geologic heterogeneity and microtopography will drive more complex patterns of drainage and flooding near the surface as well as salinity distributions in the subsurface.

The dominant mechanism of future saltwater intrusion is further identified by comparing the absolute landward movement of the saltwater toe induced by recharge reduction or sea level rise and associated coastline migration (Figure 3b). This provides a binary prioritization for the major driver at hand for saltwater intrusion on a large scale. Regions such as Australia and the East Coast of China exemplify cases where declining recharge (climatic drying) exerts the greatest control on saltwater intrusion. While groundwater pumping is not directly integrated into this analysis, dry regions should be highly susceptible to faster salinization process induced by pumping and upconing (Ferguson & Gleeson, 2012; Michael et al., 2017), as areas with drying climates may depend more heavily on groundwater to meet their water resource needs in the future (Famiglietti, 2014). Regions such as most of Southeast Asia exemplify low‐lying areas where shoreline transgression will have the greatest effect on shallow groundwater salinities, assuming no coastal defense measures are taken. In sum, our results highlight the potential pathways to adaptation and coastal defense planning that may be employed. For instance, in drying regions where declining recharge rates are expected to accelerate saltwater intrusion, it may be effective to supplement coastal adaptation measures with strategies that enhance and protect recharge. Meanwhile, low‐lying regions prone to coastal migration may prioritize shoreline protection projects or even managed retreat. The results presented here can serve as a contextual background to integrate additional complexity regarding coastal processes and anthropogenic effects. Local considerations such as pumping, geologic heterogeneity, preferential flow, vertical land motion, and episodic drivers are not captured within the model results and should be incorporated for further detail.

## 5 Summary

This work highlights the vulnerability of saltwater intrusion along the global coastline driven by the two largest climatic factors that control fresh‐saline hydrology along the land‐ocean interface. Future recharge changes drive most of the extreme cases of saltwater intrusion, while sea level rise and coastline migration drive the pervasiveness of saltwater intrusion even in regions of ample recharge. Combined, the two drivers produce spatially variable patterns of saltwater intrusion, alternating between regions that will be more influenced by one driver or the other. The results highlight the importance of integrating both future recharge and sea level changes in determining saltwater intrusion vulnerability within a coastal watershed, and provide a large‐scale context for future adaptation strategies and more complex regional studies.

## Conflict of Interest

The authors declare no conflicts of interest relevant to this study.

## Supporting information

Supporting Information S1

## Data Availability

The processed data used for saltwater intrusion calculations in the study, as well as outputs and the code are available at the HydroShare repository via Adams (2024) with a CC BY‐NC‐SA license.
