# Supplementary material for: Climate‐Induced Saltwater Intrusion in 2100: Recharge‐Driven Severity, Sea Level‐Driven Prevalence
Source: Geophys Res Lett. 2024 Nov 22;51(22):e2024GL110359. doi: 10.1029/2024GL110359 (PMC11583115; doi:10.1029/2024GL110359)
Supplement: Supplementary file 1 — Supporting Information S1 [file GRL-51-0-s001.pdf]

Geophysical Research Letters

Supporting Information for

Climate-induced saltwater intrusion in 2100: recharge-driven severity, sea level-driven prevalence

Kyra H. Adams[1]*; J.T. Reager[1]; Brett A. Buzzanga[1]; Cédric H. David[1]; Audrey H. Sawyer[2]; Benjamin D. Hamlington[1]

[1]Jet Propulsion Laboratory at California Institute of Technology, Pasadena, CA, USA., [2] School of Earth Sciences, Ohio State University, Columbus, OH, USA.

Contents of this file

Text S1: Boundary conditions
Text S2: Coastline migration
Text S3: Uncertainty analysis using Monte Carlo simulations
Figures S1 to S5
Table S1
Data: Saltwater intrusion results for Case A, B, and C

Text S1: Coastline Migration

The coastline is defined as the point of intersection between land surface and sea level, where x = 0 (Fig. 1). However, with rising sea level, surface inundation will allow inland migration of the coastline, shifting the reference point for (1), which adds to the predicted translation of the toe of the saltwater wedge. This surface inundation and coastline migration also effectively decreases the drainage length D_L (Fig. S2), decreasing the received recharge post-sea level rise and exacerbating saltwater intrusion (Fig. S5). For steady-state calculation purposes, we assume we assume that salinities in the subsurface are in equilibrium with prescribed sea level and recharge conditions (in other words, the adjustment of the freshwater-saltwater interface keeps up with shoreline migration and changes in sea level or recharge).

We assume a planar-sloping land surface from sea level (elevation = 0) to the elevation of the catchment centroid (ζ_c), obtained from SRTM-based HydroSHEDS. Then, the movement of the coastline (or coastline migration M) is calculated using simple trigonometry, where $\theta = \tan(\zeta_c / D_L)$ is the slope of the land surface:

$$\mathrm{M} = \frac{SLR}{\tan\theta}$$

Drainage length is the defined as the characteristic distance from the landward hydraulic divide (landward edge of the coastal watershed) to the coast, and is obtained by dividing the area of the coastal watershed by the length of the coastline (Figure S2).

The above equation to calculate M assumes that the freshwater-saltwater boundary follows the migration of the coastline along a planar sloping land surface, and does not account of any effects of variable density, changes to freshwater discharge, or sea level rise that are included already in the saltwater toe (x_t) equation. Thus, the component of coastline migration that represents movement of the shoreline reference frame is added, allowing it to be modified to:

$$x_{t1} = D_{L1} - \sqrt{(D_{L1})^2 - \frac{K(1+\alpha)z_1^2}{R(\alpha^2)}} + M$$

Where D_{L1} represents the shortened drainage length. This is analogous to the approach presented by Ataie-Ashtiani et al. (2013). The total movement of x_t is taken to be the difference between the original x_{t0} and the newly calculated x_{t1}. For regions such as Southeast Asia, where changing climatology drives seaward saltwater retreat in similar magnitudes as sea level rise-driven coastline migration, inundation may be the more prominent process that threatens coastal habitats.

Text S2: Boundary conditions

Saltwater intrusion progresses under two possible boundary conditions: one in which the terrestrial water table is able to rise synchronously with sea level rise ("recharge-limited"), and one in which topographic limits prevent water table rise and in turn decreases coastal freshwater discharge to accommodate sea level rise (Fig. S3). This latter boundary condition is referred to as the "topography-limited" case and is responsible for a more landward lateral intrusion of the saltwater wedge compared to the former condition(Michael et al., 2013; Werner & Simmons, 2009). Global coastal watersheds were classified into the two boundary conditions by comparing the mean elevation of the coastal watershed to projected water table rise at the centroid of the watershed after sea level rise, where estimated hydraulic head at the centroid, h_c, is given by:

$$h_c = \sqrt{(\frac{2}{K}(x - x_{t1})\left(q_0 - \frac{R}{2}(x + x_{t1})\right) + (h_t + z_1)^2)} - z_1 \quad (3)$$

where x is the distance to the centroid of the watershed from the coastline (x = 0; point of intersection between topography and sea level), z_1 the new sea level height above the base of the aquifer after a rise in sea level, and $h_t = \frac{z_0}{a}$ (head at the wedge toe). We then compared the estimated hydraulic head at the catchment centroid to mean topographic elevation across the catchment from HydroSHEDS. If elevation was higher than *h*, we assumed there was ample space in the unsaturated zone to accommodate the rising water table (Fig. S3, Recharge-limited case). The change in the saltwater toe position, x_t, with sea level rise was then calculated according to (1) using z_1 in lieu of z_0. Freshwater flux to the coast remains constant, as the slope of hydraulic gradient from the groundwater divide to the coast remains constant.

On the contrary, if average catchment elevation was lower or equal to the estimated head, h_c, the coastal watershed was regarded to be "topography-limited" (Extended Data Fig. 3). When the unsaturated zone is limited by topography ("topography-limited"), the water table cannot rise freely with sea level because it intersects the land surface, groundwater drains to area surface water bodies, and both recharge and fresh groundwater discharge are reduced. In this case, the water table position is assumed to remain constant. For such cases, Equations (1) and (3) were solved to obtained new numbers for reduced freshwater discharge and the corresponding x_t following sea level rise.

The newly calculated saltwater toe position x_{t1} was compared to the original x_{t0} to evaluate the lateral saltwater wedge movement. Sensitivity analyses for each coastal watershed with the given set of parameters were conducted to ensure that conditions did not destabilize the model and drive the saltwater toe beyond the inland no-flow boundary. For example, we only considered passive saltwater intrusion (Werner, 2017), where there is still fresh discharge directly to the ocean (opposing freshwater and seawater gradients). Watersheds with active saltwater intrusion, where fresh discharge is below the threshold and both freshwater and seawater gradients are landward, were considered to be fully intruded to the inland watershed boundary. Coastline migration, introduced for model cases with sea level rise (e.g., Case A did not have a coastline migration component as only recharge was changed), is discussed in the following section.

Text S3: Uncertainty analysis using Monte Carlo simulations

Saltwater intrusion sensitivity to hydraulic conductivity, aquifer thickness, seawater density, recharge, and sea level rise was quantified using 100,000 runs of Monte Carlo simulations for each watershed. Readers are directed to Werner & Simmons (2009) and Michael et al. (2013) to understand the general sensitivity of saltwater intrusion to recharge, sea level rise, thickness, and hydraulic conductivity. Seawater density and the resulting density ratio between fresh and saline water α (alpha) plays a relatively minor role compared to other parameters. The nominal range of α is assumed to be between 33 and 50 for typical fresh-saline variable density settings (Werner & Simmons, 2009), and the value of 40 is commonly used. The range of coastal water density derived from the ECCO v4r4 dataset was 1.022 to 1.027 g/cm^3, corresponding to a resulting α range of 37 to 45. This results in a net saltwater intrusion difference of only 0.24 m, when values for recharge, sea level rise, hydraulic conductivity, and thickness are fixed at the global mean and not varied.

The ranges of parameter values used for the Monte Carlo simulations were first defined using the standard deviation of each parameter across all global watersheds. For all parameters, a uniform distribution was used, except for hydraulic conductivity and aquifer thickness, for which log normal distributions were used. Then, 100,000 realizations of saltwater toe movement (x_{tmc}) were performed for each coastal watershed by randomly selecting combinations of parameters from the distributions. Finally, the standard deviation of x_{tmc} was calculated for each watershed (Figure 3A). The correlation coefficient quantifying the linear dependence between parameters and the standard deviation of x_{tmc} are presented in Table S1.

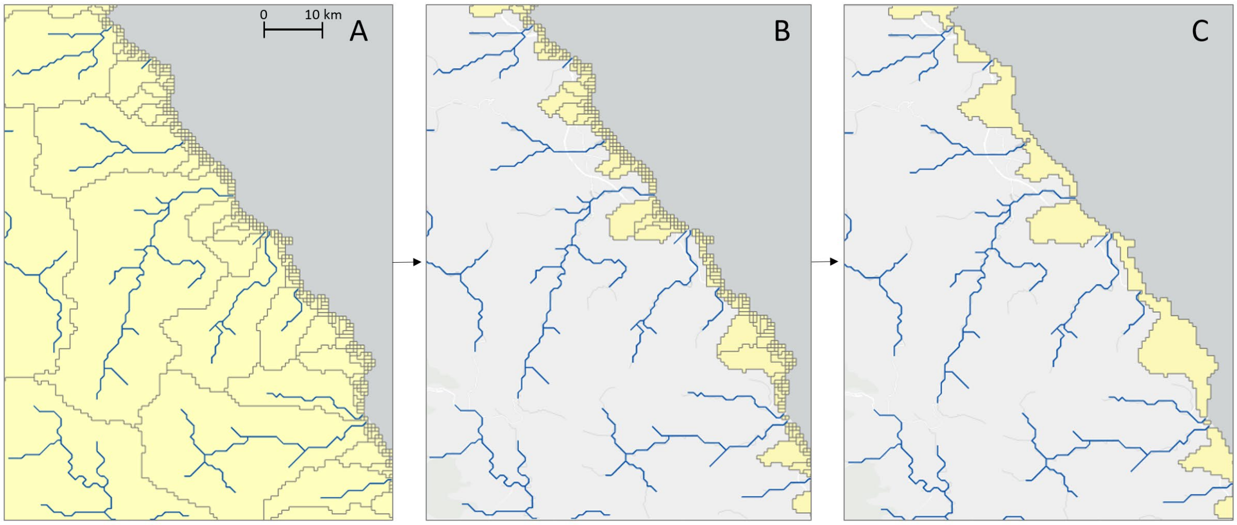

Fig. S1 Schematic diagram of coastal watershed delineation process using HydroSHEDS. HydroSHEDS is a high-resolution (approximately 500 m), near-global map of rivers and their catchments with coverage between 60°N and 60°S, based on elevation data obtained from the NASA Shuttle Radar Topography Mission (SRTM) (Farr et al., 2007). **A:** 30 arc-second resolution HydroSHEDS basin outlines, based on NASA's Shuttle Radar Topography Mission, are collocated with surface water channels. **B:** Coastal watersheds between larger inland, surface water-containing watersheds are selected. **C:** The watersheds are dissolved into meaningful watershed delineations using the catchment separation points as nodes. Within the coastal catchments, no surface water streams are included.

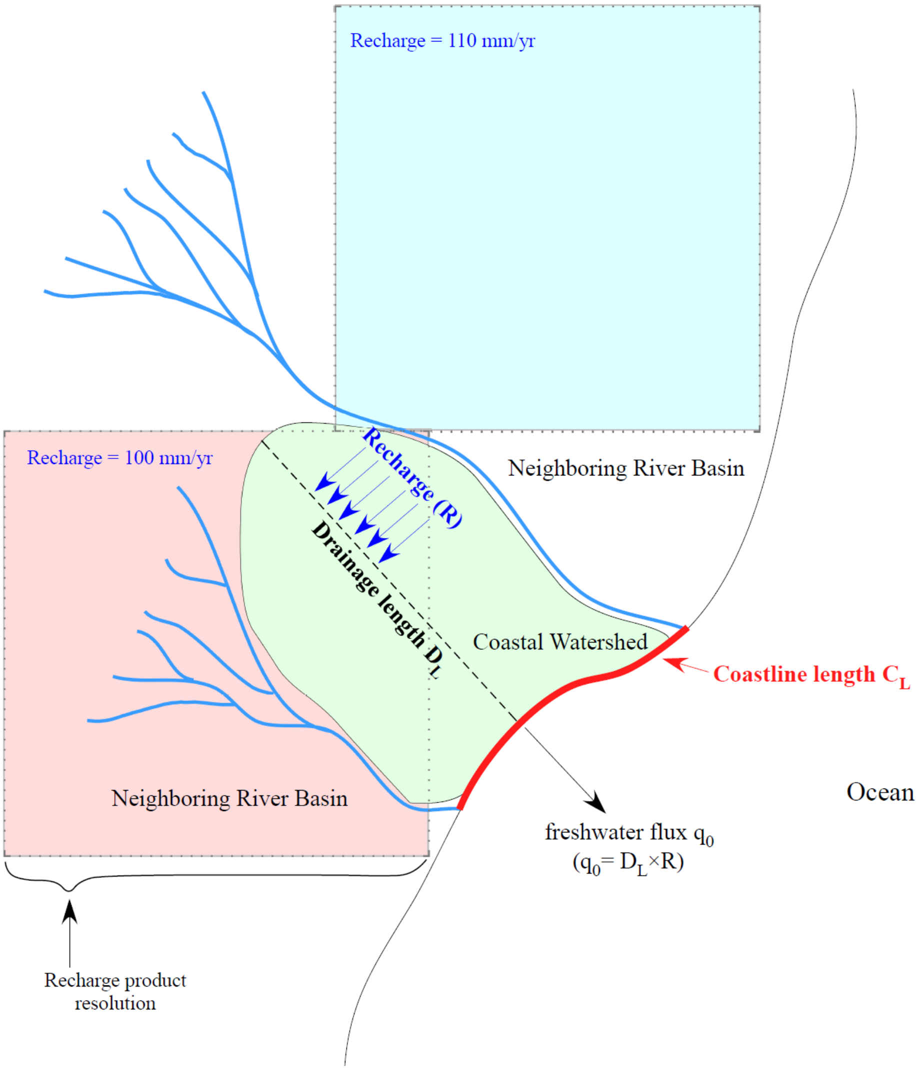

Fig. S2 Schematic diagram showing the method by which freshwater discharge q_0 was calculated using recharge rate R and watershed geometry. The nearest pixel to the centroid of the coastal watershed was selected from the recharge product (GLDAS or ISIMIP) to get the recharge rate. Assuming that the inland watershed boundary represents a hydraulic divide (no lateral flux into the watershed) the total fresh discharge q_0 per unit length of shoreline is $\mathbf{R} \times \mathbf{Area} \div \boldsymbol{C_L}$ or $\boldsymbol{R} \times \boldsymbol{D_L}$ if area is represented by $\boldsymbol{C_L} \times \boldsymbol{D_L}$.

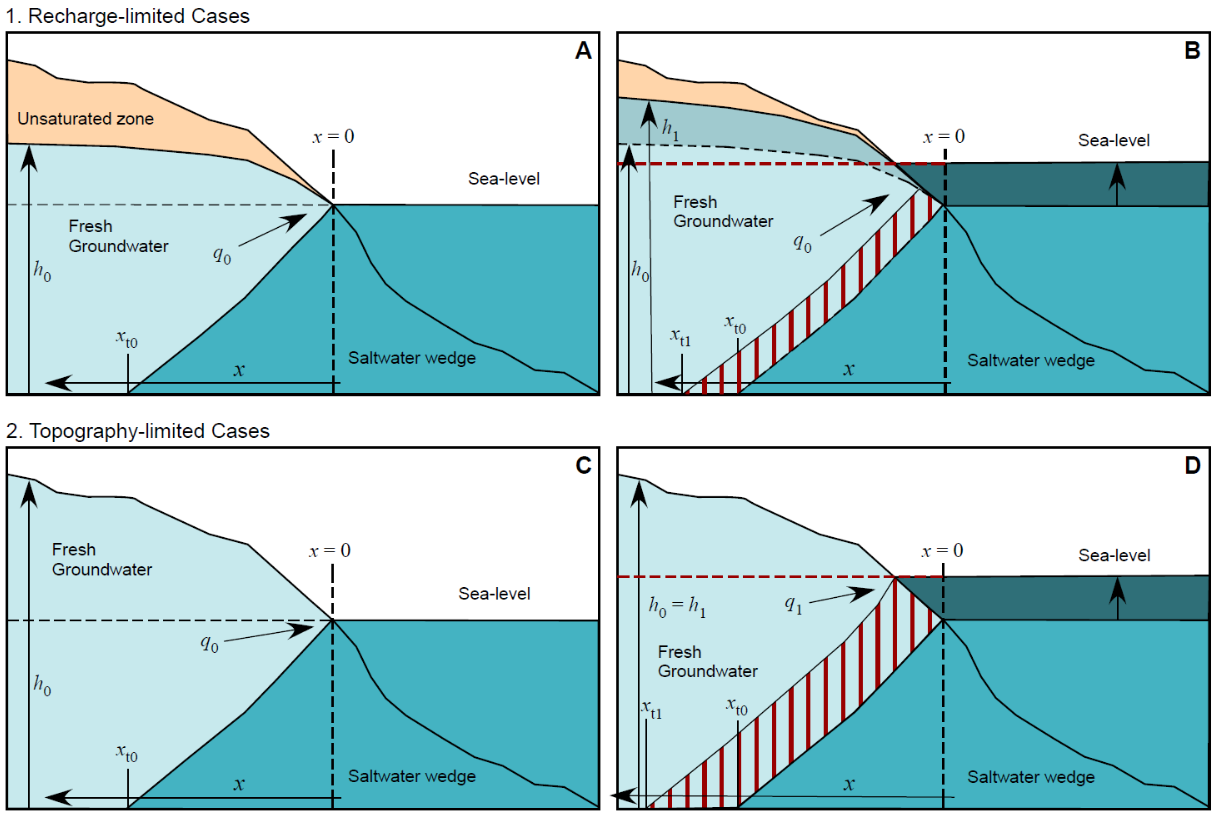

Fig. S3 The two types of boundary conditions. Sea level rise and lateral saltwater intrusion shown in red (B, D). **Top:** 1. Recharge-limited cases have ample unsaturated zone to allow groundwater head to rise (B, h_0 to h_1) with sea level (B, red). Submarine Groundwater Discharge flux is therefore constant and saltwater intrusion is combatted. **Bottom:** 2. Topography-limited cases do not allow head rise, decreasing hydraulic gradient as sea level rises and consequently, the flux of fresh discharge (D, q_0 *to* q_1). Compared to the constant flux case, there is more saltwater intrusion.

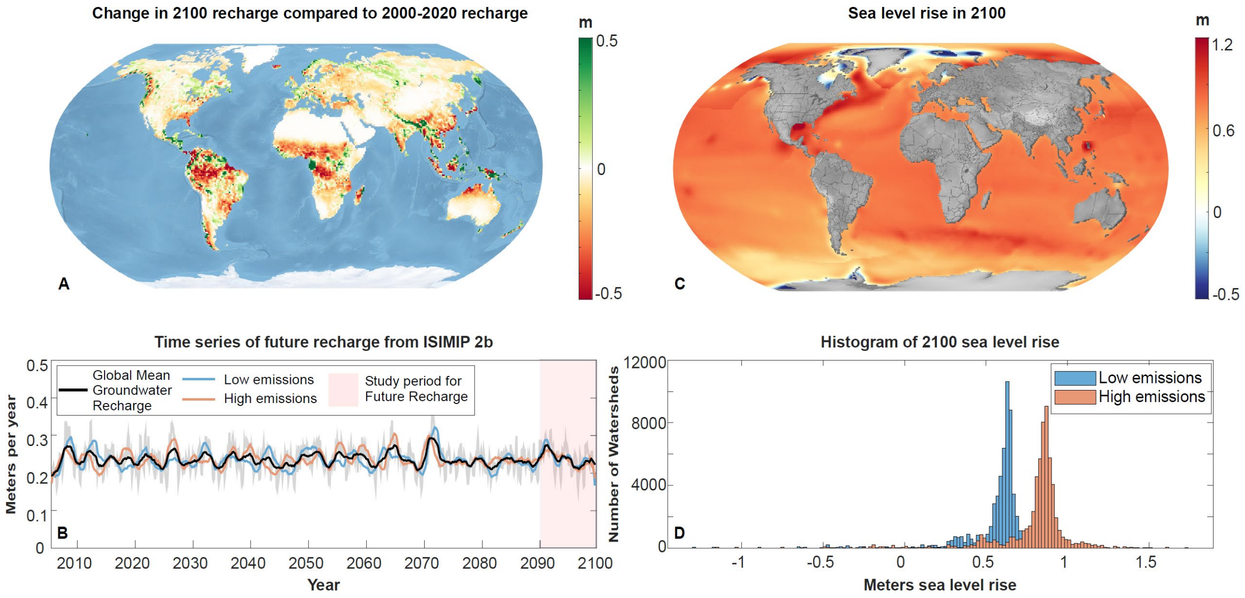

Fig. S4 Input conditions for calculating 2100 x_{toe}. A: Map of changes to recharge in 2100 obtained from ISIMIP 2b outputs, compared to 2000-2020 mean recharge from GLDAS. B: Timeseries of recharge for both the low-emissions IPCC RCP 2.6 scenario (blue) and the high-emissions IPCC RCP 8.5 (orange) scenario. The mean of two emissions scenarios (black) is used as the input. C: Future relative sea level rise, mean of the two IPCC RCP 2.6 and IPCC RCP 8.5 scenarios. D: Histogram of 2100 sea level rise experienced by global watersheds for both IPCC RCP 2.6 and IPCC RCP 8.5 scenarios.

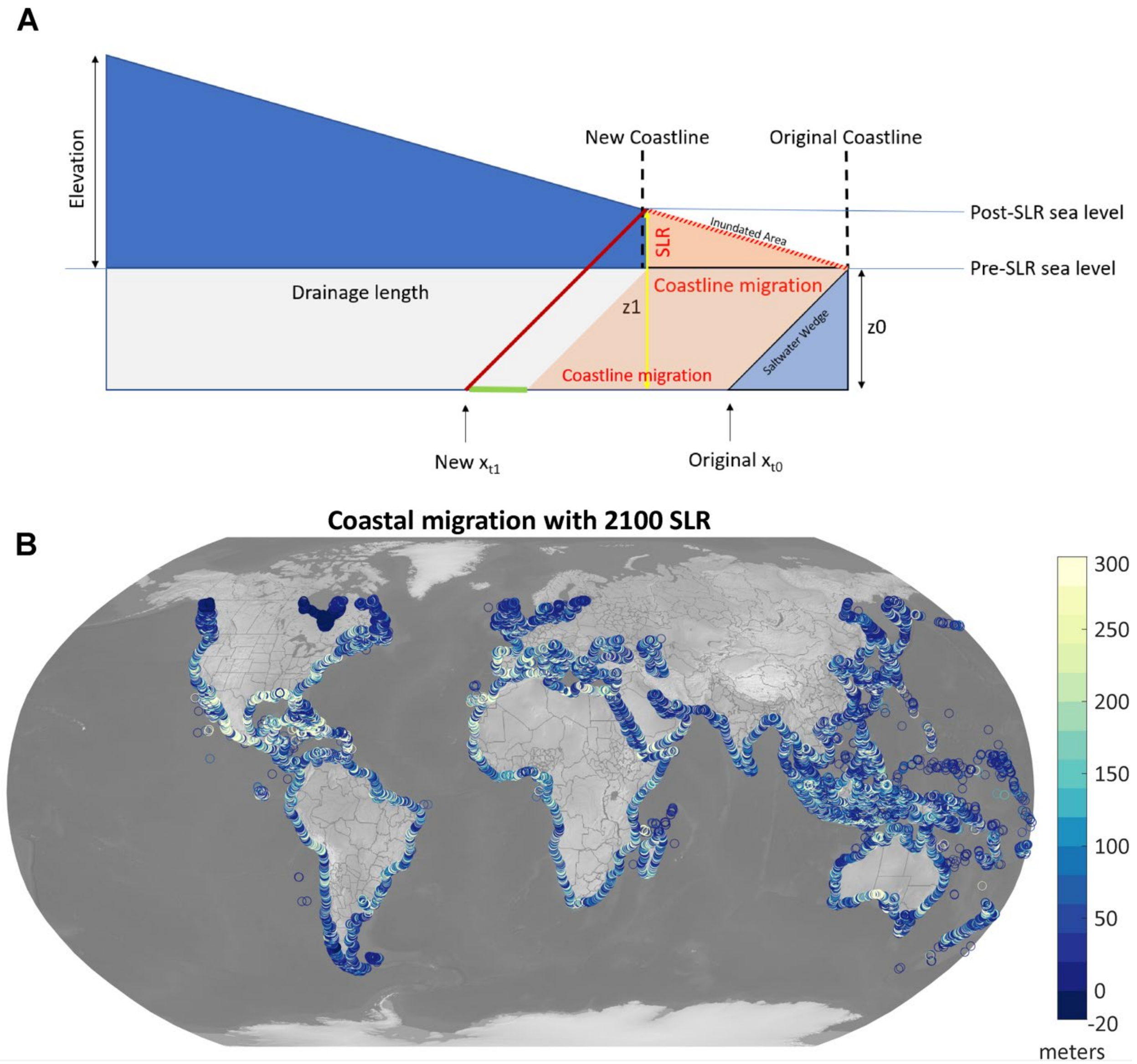

Fig. S5 Schematic diagram of coastline migration. A: Theoretical aquifer cross-section of an aquifer with coastline migration. Original coastline is at the righthand boundary. With sea level rise and inundation (orange triangle and hatched line), we assume the original x_t also moves to follow the landward migration of the coastline. The change in x_t due to the saltwater height (z_1) itself, driven by variable-density, is calculated by Equation (1) (green). δx_t is taken to be the difference between x_{t0} and $x_{t1,}$ where x_{t1} includes both coastline migration and equation-calculated intrusion.

Parameter	Hydraulic Conductivity	Aquifer Thickness	Density Ratio	Recharge	Sea Level Rise
Test range	(logarithmic)<br>± $10^{1.16}$ m/year	(logarithmic)<br>± $10^{0.38}$ m	± 2.88	± 0.69 m/year	± 0.21 m
Correlation coefficient with x_{tmc}	0.44	0.33	0.01	-0.10	0.03

Table S1. Correlation coefficients between x_{tmc} and other parameters.

Data: Saltwater intrusion results for Case A, B, and C

The processed data used for saltwater intrusion calculations in the study, as well as outputs and the code are available at the HydroShare repository “1D-analytical framework for saltwater intrusion analysis” via https://doi.org/10.4211/hs.65ccb3e358834424a2a7204b99bcc642 with a CC BY-NC-SA license.
